# Systemic Associations with Residual Subretinal Fluid after Ranibizumab in Diabetic Macular Edema

**DOI:** 10.1155/2017/4834201

**Published:** 2017-07-27

**Authors:** Meng-Ju Tsai, Yi-Ting Hsieh, Elizabeth P. Shen, Yi-Jie Peng

**Affiliations:** ^1^Department of Ophthalmology, Taipei Tzu Chi Hospital, Buddhist Tzu Chi Medical Foundation, New Taipei, Taiwan; ^2^Department of Ophthalmology, National Taiwan University Hospital, Taipei, Taiwan

## Abstract

**Purpose:**

To investigate the impact of systemic diseases on the occurrence of subretinal fluid (SRF) in diabetic macular edema (DME) and prognostic factors for residual SRF following three consecutive monthly intravitreal ranibizumab.

**Methods:**

Ninety-seven eyes from 68 patients with DME who completed 3 consecutive monthly injections of ranibizumab were enrolled. Systemic parameters mainly included chronic kidney disease (CKD), hypertension, HbA1c, and insulin dependence. Renal parameters for CKD were serum creatinine, estimated glomerular filtration rate (eGFR), and serum albumin. Ocular factors were baseline central macular thickness (CMT), severity of diabetic retinopathy (DR), and status of panretinal photocoagulation (PRP).

**Results:**

Chronic kidney disease had significant correlation with baseline SRF (*R* = 0.397, *p* < 0.001 after partial correlation with adjustment for age and DR severity). As for CKD, lower serum albumin, but not eGFR or serum creatinine, was associated with baseline presence of SRF (*p* = 0.026, *p* = 0.08 and *p* = 0.53, resp., after adjustment for age and DR severity). Overall, lower eGFR and lower HbA1c values, contrary to popular belief, predicted the presence of residual SRF following intravitreal injections (*p* = 0.016 and *p* < 0.001, resp.).

**Conclusions:**

Tight sugar control and poorer baseline kidney function may slow the resorption of SRF after anti-VEGF injections in patients with DME in the short term.

## 1. Introduction

Diabetic macular edema has been one of the leading causes of visual impairment worldwide [1]. The pathogenesis has been linked to the breakdown of blood retinal barrier induced by oxidative stress from sustained hyperglycemia and accumulation of inflammatory cytokines as well as vascular endothelial growth factors (VEGF) [2, 3]. In recent years, anti-VEGF agents have been the mainstay of treatment based on these considerations [4]. Ranibizumab, a humanized monoclonal antibody that targets intraocular VEGF, is quite effective and safe in the resolution of diabetic macular edema reported by several studies [4, 5]. Subretinal fluid was found to be a favorable indicator for both visual and anatomical improvement following ranibizumab treatment [6]. Presence of subretinal fluid was more likely to achieve final central macular thickness 250 *μ*m or less, and vision 20/40 or better. However, if left untreated, persistent subretinal fluid may be detrimental to photoreceptors and retinal pigmented epithelium (RPE) [7, 8]. Mechanisms for subretinal fluid in DME remained to be elucidated, including insufficient fluid removal by impaired RPE pumping [9], and disruption of external limiting membrane causing fluid shift from intraretinal space to outer retina [10, 11]. Other studies also observed the increased levels of intraocular VEGF which may contribute to the level of fluid as evidenced by optical coherent tomography reflectivity [12]. A few studies also reported the association of subretinal fluid with chronic kidney disease [13, 14]. Diabetic nephropathy often coexists with advanced diabetic retinopathy, suggesting that they may share similar microvascular pathophysiology [15]. The purpose of the study is to investigate the role of kidney function in the subretinal fluid of diabetic macular edema, and correlating factors for residual fluid following three monthly ranibizumab treatments.

## 2. Materials and Methods

Ninety-seven eyes from 68 patients were retrospectively enrolled according to medical records from October 2012 to July 2016 in Taipei Tzu Chi Hospital. All eyes with diabetic macular edema had completed three consecutive monthly intravitreal injections of ranibizumab with follow-up at one month after each injection. Inclusion criteria were macular edema evidenced by initial CMT value of 300 *μ*m or more. Macular edema of other causes such as uveitis and retinal vein occlusion was excluded. Eyes that underwent previous intraocular surgery or intravitreal injection of any drug within 3 months prior to the inclusion were also excluded. This research adhered to the tenets of the Declaration of Helsinki, and Institutional Review Board (IRB) approval was obtained from the IRB of Taipei Tzu Chi Hospital, Buddhist Tzu Chi Medical Foundation.

### 2.1. Data Collection

The presence of subretinal fluid was defined as an anatomical separation between neurosensory retina and retinal pigmented epithelium under the fovea. Both subretinal fluid and central macular thickness was documented by OCT (Cirrus HD-OCT 400, Carl Zeiss Meditec, Dublin, CA, USA) at baseline and 3 months after ranibizumab injections. The presence of SRF in each eye was deemed by different surgeons. The interobserver agreement for SRF was validated in the previous study [16]. As to blood test, serum creatinine, glycosylated hemoglobin, and albumin levels were checked prior to first ranibizumab injection. Chronic kidney disease was defined according to KDOQI (Kidney Disease Outcomes Quality Initiative) guideline. Baseline estimated glomerular filtration rates were categorized into three groups, eGFR less than 30 (stage 1), between 30 and 60 (stage 2), and 60 or more (stage 3).

### 2.2. Statistical Analysis

For continuous variables such as age, CMT, and HbA1c levels, GLM/ANOVA analysis was performed for comparison among groups with different kidney functions, and nonparametric Mann–Whitney *U* test was used to examine the difference in renal parameters in chronic kidney disease. Discrete variables were analyzed by chi-square test or Fisher's exact test. Spearman's correlation was used to evaluate the correlation of baseline subretinal fluid to clinical parameters, and partial correlations were also performed to adjust for age and DR severity. To take into account of correlations between 2 eyes from 1 subject, binary logistic regression models with generalized estimating equation were used to determine the prognostic factors for presence of residual subretinal fluid after 3 injections. SPSS for Windows (Version 18; SPSS Inc., Chicago, IL, USA) was used for statistical analysis. A *p* value less than 0.05 was considered statistically significant.

## 3. Result

### 3.1. Baseline Demographics

**Figure 1:**
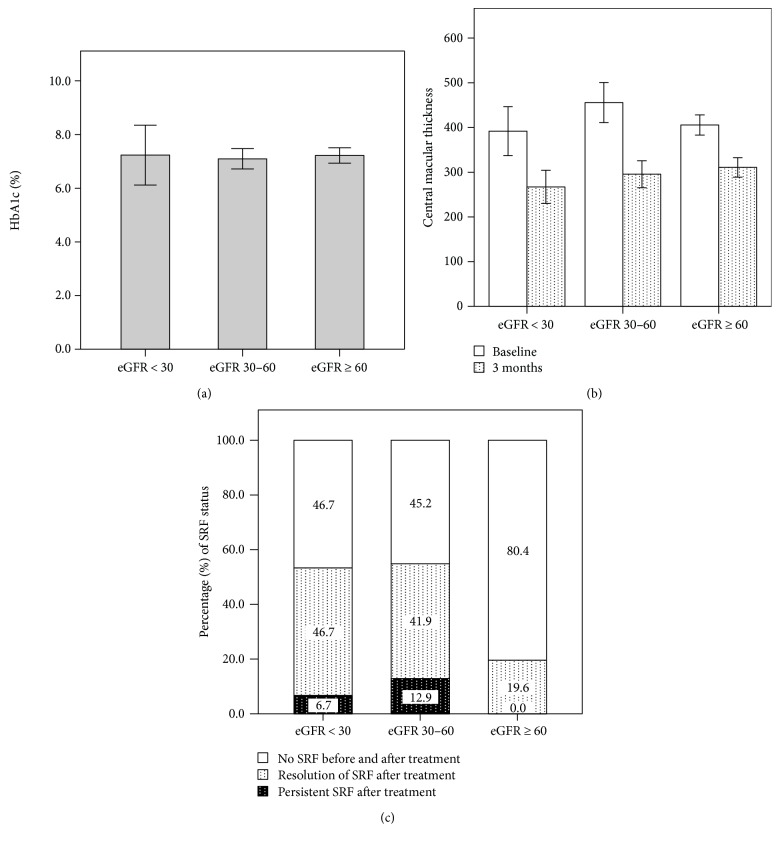
Presentation of clinical parameters in relation to different kidney functions with eGFR < 30, between 30 and 60, and ≥60. The differences among the three groups were not statistically significant in (a) HbA1c level (*p* = 0.90, GLM/ANOVA) and (b) central macular thickness before and after treatment (*p* = 0.051 and 0.17, resp., GLM/ANOVA). Data were in mean ± 2SE. In (c), poorer kidney function was associated with the presence of subretinal fluid before and after treatment (*p* = 0.001 and 0.04, resp., Spearman's correlation).

A total of 97 eyes from 68 patients were enrolled in this study. Fifty-four patients (79.4%) had PDR and 33 (48.5%) had chronic kidney disease. Subretinal fluid was presented in 25 patients (36.8%) before treatment. Overall, mean age was 61.5 ± 9.0 years, average glycosylated hemoglobin level was 7.2 ± 1.3%, and mean baseline central macular thickness was 419 ± 102 *μ*m. Data were further categorized according to different kidney functions (Figure 1).

### 3.2. Subretinal Fluid with Systemic Correlations

**Figure 2:**
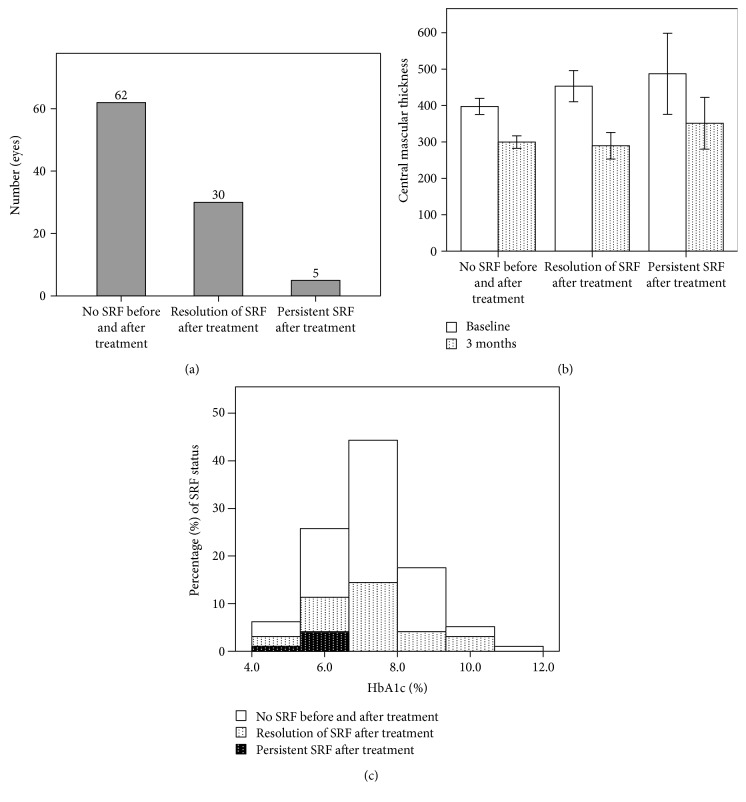
Presentation of clinical parameters according to different status of subretinal fluid. (a) Number of eyes with absence of SRF before and after treatment, resolution of initial SRF after treatment, and persistent SRF after treatment. (b) Central macular thickness before and after treatment and (c) HbA1c levels.

The correlations of clinical parameters to baseline subretinal fluid were presented in Table 1. There were no significant correlations of baseline SRF with age, baseline CMT, HbA1c, DR severity, PRP status, hypertension, or insulin dependence. Subretinal fluid had significant correlation with chronic kidney disease, and partial correlation of CKD with SRF was still significant after adjustment for age and DR severity (*R* = 0.397, *p* < 0.001).  Table 2 depicted the ocular characteristics in relation to different kidney functions. Lower levels of eGFR were markedly associated with the presence of SRF before treatment, 53.3% and 54.8% of eyes in eGFR below 30 and eGFR between 30 and 60, respectively, and 19.6% in eGFR 60 or more. After 3 ranibizumab injections, 30 of these 35 eyes had resolution of subretinal fluid, while the other 5 eyes had persistent fluid (Figure 2(a)). Characteristics of these 5 eyes were presented in Table 3. Distribution of subretinal fluid before and after treatment according to varying baseline HbA1c levels was shown in Figure 2(c).

### 3.3. Subretinal Fluid with PDR/NPDR before Treatment

Before treatment, PRP naïve PDR had a greater proportion of subretinal fluid than eyes with NPDR (*p* = 0.028, Fisher's exact test). In PDR, baseline subretinal fluid was associated with the timing of PRP (*R* = −0.356, *p* = 0.002). PRP naïve eyes had the highest percentage of baseline subretinal fluid before treatment, 61.9%, compared with 42.9% and 20.6% in PRP within 6 months and beyond 6 months, respectively.

### 3.4. Subretinal Fluid with Renal Parameters in CKD

Presence of SRF before treatment had lower serum albumin values (*p* = 0.017) and higher eGFR (*p* = 0.043), while serum creatinine was not significantly different between presence and absence of SRF in CKD (*p* = 0.13) (Table 4).

### 3.5. Prognostic Factors for Residual SRF after Ranibizumab

Subretinal fluid resolved in all cases after ranibizumab treatment except 5 eyes (5.2%). Lower baseline HbA1c and lower eGFR stages were more likely to result in residual SRF at 3 months. Age, insulin dependence, baseline CMT, and status of PRP did not affect final SRF status (Table 5).

## 4. Discussion

Our data suggested that chronic kidney disease played a role in the occurrence of subretinal fluid in diabetic macular edema. Presence of subretinal fluid before treatment had good correlations with lower eGFR stages and lower albumin levels. To date, the reason why subretinal fluid only appears in a small portion of diabetic macular edema has remained unclear. High glycosylated hemoglobin levels and systemic hypertension have been linked to the breakdown of blood retinal barrier that makes fluid tend to shift into the subretinal space [17, 18]. High glucose levels increased osmotic and oxidative stress which impaired RPE pumping function as suggested in the previous study [17]. We did not directly observe the correlations; however, this may not be comparable due to variations of disease severity in the eyes included. The previous study had much higher average HbA1c, 10.05% in SRF group, compared with 7.0% in our study. In addition to RPE dysfunction, literatures had pointed out that prolonged hyperglycemia and hypertension could directly or indirectly contribute to upregulation of vascular endothelial growth factors due to sustained ischemia and RPE injury [18–20]. One clinical research showed in 15 eyes that subretinal fluid reflectivity quantified by OCT was highly associated with increased levels of intraocular VEGF that led to the breakdown of blood retinal barrier [12]. One report showed the evidence that elevated serum VEGF in patients with diabetic retinopathy had correlation with severity of disruption of external limiting membrane (ELM) [21], which serves as a barrier to subretinal space. Damage to the ELM may lead to accumulation of proteins and fluid in the subretinal space. The buildup of vascular endothelial growth factors in the formation of SRF was hence evidenced by the consistent findings. Chronic kidney disease might be a systemic source of VEGFs, which can be produced in a compensatory way by glomerular podocytes and tubular epithelial cells in response to nephron loss. Numerous experimental models had strongly revealed the evidence of overexpression of VEGF mRNA implicated in the pathogenesis of diabetic nephropathy, particularly at the early stages [22–25]. Elevated serum VEGF has been testified to be an early indicator for progression of diabetic nephropathy [23], giving an explanation why subretinal fluid tends to occur in chronic kidney disease with relatively well-preserved kidney function that additionally contributed to intraocular VEGF levels.

In chronic kidney disease, serum albumin was a sensitive marker for the presence of subretinal fluid. Hypoalbuminemia indicated severe loss of albumin as seen in cases with nephrotic syndrome. In contrast to VEGF that causes vascular hyperpermeability and breakdown of blood retinal barrier, decrease in serum albumin may lower the intravascular osmotic pressure and increase hydrostatic pressure that leads to fluid retention in the third space, including subretinal space [14]. Basically, resorption of subretinal fluid depends on both passive diffusion and active pumping by retinal pigmented epithelium as previously mentioned [9], yet this may be impaired by sustained hypoxia in the setting of severe diabetic retinopathy. A few case series with exudative neurosensory detachment were reported secondary to hypoalbuminemia from nephrotic syndrome with or without diabetes mellitus, and the subretinal fluid in these cases resolved following systemic furosemide treatment [14]. These findings add to the complexity of subretinal fluid that reflects more than merely the ocular morbidity.

Opposite to the general belief, we observed that lower baseline HbA1c levels were more likely to result in residual SRF following 3 monthly intravitreal ranibizumab. While no such conclusion has been drawn from the current studies, there are some possible reasons to explain. Similar to early worsening effect mentioned in the previous literature [26, 27], this phenomenon could be caused by intensive glycemic control with rapid reduction of HbA1c levels. In this setting, the worsening phenomenon may be attributed to decreased nutrient subtract and the ability of the retinal circulation to autoregulate [26]. It is worth mentioning that the DCCT included patients with much milder degrees of retinopathy or even no retinopathy, which is different from the severity in the present study. Theoretically, the early worsening phenomenon could be transient if retinal endothelial cells are not severely damaged and regain their physiologic functions to maintain blood retinal barrier. In the context of severe DR with macular edema as seen in our study, we inferred that the early worsening may become prolonged if the cells irreversibly lose the abilities to autoregulate to adapt back to relatively hypoglycemic state. Data from the more recent study have also given the supportive hint that in macular edema, better control of HbA1c did not lead to greater reduction of central macular thickness [28]. The post hoc analysis of RISE and RIDE showed the association between smaller percentage of CMT resolution to less than 250 *μ*m and improved HbA1c at 36 months (*p* = 0.04).

On a molecular basis, the application of Starling's rule may also provide us some insights to understand how fluid can shift across between two spaces with pressure gradient created by osmotic and hydrostatic pressure [29]. In the setting of disrupted retinal barrier, increased interstitial osmotic pressure caused by extravasations of various proteins and molecules drained fluid into the inner and outer retina. Lowering of serum glucose by preceding tight glycemic control, however, lowers the intravascular osmotic pressure. The pressure gradient increased the tendency to move the fluid from intravascular to retinal parenchyma. We inferred that pressure gradient between outer retina and subretinal space, or at the level of RPE, played a role in directing the fluid movement by a similar mechanism as Starling's rule has implied. Fluid with higher hydrostatic pressure in outer retina or choroidal vessels may then flow into subretinal space more readily to reduce higher osmolality. The effect of osmotic difference between subretinal space and systemic osmolality had been demonstrated to affect fluid resorption in an animal model [30]. This passive diffusion at the level of RPE may become even more important particularly when active pumping function of the RPE was impaired. In one basic research, the concept of Starling's rule may perhaps have a role to explain the observation that intracellular adhesion molecule-1 (ICAM-1), an immunoglobulin superfamily upregulated by VEGF, was inversely related to SRF height [31]. Another supportive evidence showed reduced central macular thickness after meal compared with that of before meal, suggesting the glucose effect on the fluid distribution across the retina [32]. In our study, all the five eyes with residual subretinal fluid in the present study had HbA1c values ranging from 4.8 to 6.6%, compared with the average 7.2%. The reconstruction of blood retinal barrier as seen in cases after ranibizumab treatment may actually reinforce the osmotic effect, which accounts for this result. One human study also provided indirect evidence that initiation of osmotic water movement from retina to blood vessels resulted in macular volume reduction [33]. The role of sugar control may become more complicated when intervention for macular edema was considered.

There are certainly limitations in our study. First, this is a retrospective short-term study with inherent selection bias and a small sample size. The small number of residual SRF may limit the power of statistical analysis to yield a robust conclusion. We found that residual SRF after treatment occupied only approximately 5.2% of a total of 97 eyes, or 14.3% of 35 eyes with SRF before treatment. This probably reflects that three ranibizumab injections are quite effective in the resolution of subretinal fluid and macular edema in most of the cases. Second, we analyzed the spot values of clinical parameters for baseline, for example, the estimated glomerular filtration rates and serum albumin levels, which may fluctuate over time. We did not obtain values at 3 months to see if these changes could have significant impact on the result. Despite these limitations, this study is of clinical significance because we highlighted some important points from systemic perspectives. Moreover, this study raises concern as to whether tight sugar control really exerts greater benefits for those with diabetic macular edema and subretinal fluid under the treatment of anti-VEGF agents. Further research should be done to clarify these mechanisms.

In conclusion, systemic diseases have significant correlations with the presence of subretinal fluid in diabetic macular edema. Chronic kidney diseases are related to the presence of initial and residual SRF after three monthly ranibizumab injections. Tight control of glycosylated hemoglobin, however, is more likely to have residual fluid possibly due to effect of osmotic balance where serum glucose plays a major role. Thus, the impact of systemic conditions should be taken into account for evaluation of response to anti-VEGF treatment.

## Supplementary Material

Supplementary Tables. Table S1. Baseline demographics in eyes with diabetic macular edema. Table S2. Correlating factors for central macular thickness after ranibizumab.

## Figures and Tables

**Table 1:** Correlations of subretinal fluid with baseline clinical parameters.

Parameters	Group 1 (*n* = 68)		Group 2 (*n* = 68)		All eyes (*n* = 97)	
	Correlation coefficient	*p* value	Correlation coefficient	*p* value	Correlation coefficient	*p* value
Age	−0.121	0.33	−0.241	0.05	−0.217	0.033
Baseline CMT	0.329	0.006	0.23	0.06	0.27	0.008
HTN	0.113	0.36	0.168	0.17	0.193	0.06
Insulin dependence	0.152	0.22	0.19	0.12	0.224	0.027
Chronic kidney disease	0.358	0.003	0.363	0.002	0.416	<0.001
HbA1c	−0.127	0.3	−0.038	0.76	−0.122	0.24
DR severity	0.237	0.05	0.006	0.96	0.12	0.24
PRP status	−0.199	0.1	−0.141	0.25	−0.171	0.1

HTN: hypertension; CMT: central macular thickness; DR: diabetic retinopathy, designated as proliferative or nonproliferative diabetic retinopathy; PRP: panretinal photocoagulation; group 1: the right eye was included if both eyes in a subject were enrolled; group 2: the left eye was included if both eyes in a subject were enrolled.

**Table 2:** Clinical parameters according to different kidney function.

eGFR (ml/min/1.73 m^2^)	<30	30–60	≥60	*p* value
Number (eyes)	15	31	51	
Age (years, mean ± SD)	61.0 ± 10.1	59.6 ± 8.4	61.2 ± 9.6	0.75
HbA1c (%, mean ± SD)	7.2 ± 2.2	7.1 ± 1.1	7.2 ± 1.0	0.90
PDR (eyes)	11 (73.3%)	27 (86.2%)	38 (75.5%)	0.55
Subretinal fluid (eyes)				
Baseline SRF (*n* = 35)	8 (53.3%)	17 (54.8%)	10 (19.6%)	0.001
SRF at 3 months (*n* = 5)	1 (6.7%)	4 (12.9%)	0 (0%)	0.04
Central macular thickness (*μ*m)				
Baseline CMT	392 ± 106	456 ± 124	406 ± 81	0.051
CMT at 3 months	267 ± 72	296 ± 84	311 ± 78	0.17

CMT: central macular thickness; PDR: proliferative diabetic retinopathy; SRF: subretinal fluid.

**Table 3:** Characteristics of eyes with residual subretinal fluid after Ranibizumab.

Number	Age	HbA1c	CMT at baseline	CMT at 3 months	DR severity	PRP status	eGFR	Serum albumin
1	62	6.3	475	361	PDR	≤6 months	59.6	3.4
2	58	6.1	392	284	PDR	>6 months	49.2	4.3
3	44	5.4	380	258	PDR	Naïve	50.4	3.2
4	61	4.8	500	414	PDR	Naïve	10.5	1.9
5	62	6.6	690	440	PDR	≤6 months	59.9	N/A

CMT: central macular thickness; DR: diabetic retinopathy, designated as proliferative or nonproliferative diabetic retinopathy; eGFR: estimated glomerular filtration rate; PDR: proliferative diabetic retinopathy; PRP: panretinal photocoagulation.

**Table 4:** Correlation of baseline status of subretinal fluid to renal parameters in chronic kidney disease.

Serum profiles	Presence of SRF (*n* = 27)	Absence of SRF (*n* = 21)	*p* value^∗^
Creatinine (mg/dL)	2.2 ± 1.6	2.7 ± 2.5	0.13
eGFR (ml/min/1.73 m^2^)	41.9 ± 15.8	33.1 ± 14.1	0.043
Albumin (g/dL)	2.7 ± 0.8 (*n* = 24)	3.3 ± 0.5 (*n* = 15)	0.017

eGFR: estimated glomerular filtration rate; SRF: subretinal fluid; ∗ denotes *p* values calculated by the Mann–Whitney *U* test.

**Table 5:** Correlating factors for residual subretinal fluid after ranibizumab.

Factor	OR for residual SRF (95% CI)	Adjusted *p* value
Age	0.917 (0.835–1.009)	0.74
Baseline CMT	1.005 (0.997–1.014)	0.218
HbA1c	0.215 (0.117–0.394)	<0.001
Insulin dependence	1.965 (0.242–15.944)	0.527
PRP status		
PRP naïve	Reference	
PRP beyond 6 months	2.692 (0.175–41.494)	0.478
PRP within 6 months	20.266 (0.908–452.283)	0.058
eGFR^∗^	0.093 (0.013–0.644)	0.016

CMT: central macular thickness; DR: diabetic retinopathy, designated as proliferative or nonproliferative diabetic retinopathy; eGFR: estimated glomerular filtration rate; PDR: proliferative diabetic retinopathy; PRP: panretinal photocoagulation; ∗ denotes eGFR values categorized to stages as described in Table 2.
