# Supplementary material for: Systemic Associations with Residual Subretinal Fluid after Ranibizumab in Diabetic Macular Edema
Source: J Ophthalmol. 2017 Jul 27;2017:4834201. doi: 10.1155/2017/4834201 (PMC5551529; doi:10.1155/2017/4834201)
Supplement: Supplementary file 1 — Supplementary Tables. Table S1. Baseline demographics in eyes with diabetic macular edema. Table S2. Correlating factors for central macular thickness after ranibizumab. [file 4834201.f1.pdf]

Supplementary tables

Table S1. Baseline Demographics In Eyes With Diabetic Macular Edema

Parameter	Group 1 (n=68)	Group 2 (n=68)	All eyes (n=97)
Age (years, mean±SD)	61.5±9.0	61.5±9.0	60.7±9.2
Baseline CMT (μm, mean±SD)	413±99	422±109	419±102
HTN (eyes, %)	56 (82.4)	56 (82.4)	79 (81.4)
Insulin dependence (eyes, %)	16 (23.5)	16 (23.5)	19 (19.6)
CKD (eyes, %)	33 (48.5)	33 (48.5)	48 (49.5)
HbA1c (%, mean±SD)	7.2±1.3	7.2±1.3	7.2±1.3
DR severity (eyes, %)			
NPDR	14 (20.6)	15 (22.1)	21 (21.6)
PDR	54 (79.4)	53 (77.9)	76 (78.4)
PRP status (eyes, %)			
PRP naïve	29 (42.6)	30 (44.1)	42 (43.3)
PRP within 6 months	15 (22.1)	15 (22.1)	21 (21.6)
PRP beyond 6 months	24 (35.3)	23 (33.8)	34 (35.1)

HTN: hypertension, CKD: Chronic kidney disease, CMT: central macular thickness, DR: diabetic retinopathy, designated as PDR or NPDR

Group 1: Right eye included if both eyes in a subject were enrolled

Group 2: Left eye included if both eyes in a subject were enrolled

Table S2. Correlating Factors For Central Macular Thickness After Ranibizumab

Factor	Beta Coefficient	Adjusted P value
Age	0.881	0.33
Baseline CMT	0.163	0.02
HbA1c	-2.432	0.702
Insulin Dependence (Reference: insulin-)	25.108	0.16
PRP status		
PRP beyond 6 months	36.015	0.054
PRP within 6 months	56.83	0.007
(Reference: PRP naïve)		
eGFR*	21.852	0.039

eGFR* denotes values categorized to stages as described in Table 2
